# Supplementary material for: Integrative Analysis of Gene Expression and Promoter Methylation to Differentiate High-Grade Serous Ovarian Cancer from Benign Tumors
Source: Biomedicines. 2025 Feb 11;13(2):441. doi: 10.3390/biomedicines13020441 (PMC11853219; doi:10.3390/biomedicines13020441)
Supplement: Supplementary file 1 [file biomedicines-13-00441-s001.zip › biomedicines-3452129-supplementary.pdf]

Integrative Analysis of Gene Expression and Methylation to Differentiate High-Grade Serous Ovarian Cancer from Benign Tumors

Ieva Vaicekauskaitė[1,3], Paulina Kazlauskaitė[2,3], Rugilė Gineikaitė[1], Rūta Čiurlienė[4], Juozas Rimantas Lazutka[1], Rasa Sabaliauskaitė[1,3]

[1] Institute of Biosciences, Life Sciences Center, Vilnius University, Vilnius, Lithuania;

[2] Institute of Biomedical Sciences, Faculty of Medicine, Vilnius University, Vilnius, Lithuania;

[3] National Cancer Institute, Vilnius, Lithuania;

[4] National Cancer Center, Vilnius University Hospital Santaros Klinikos, Vilnius, Lithuania.

*** corresponding author:** Rasa Sabaliauskaitė, PhD; Life Sciences Centre, Vilnius University, Sauletekio al 7, 10257 Vilnius, Lithuania; e-mail: rasa.sabaliauskaitė@gmc.vu.lt

Supplementary materials

Supplements Table S1 Primer sequences used in RT-qPCR and MSP experiments.

Gene expression primers

**Gene**	**Primer**	**Sequence (5‘→ 3‘)**
JAG2	Forward	GCTGCTACGACCTGGTCAATGA
	Reverse	AGGTGTAGGCATCGCACTGGAA
DLL1	Forward	GGGTCATCCTTGTCCTCAT
	Reverse	CTTGGTGTCACGCTTGCT
HES1	Forward	ACGTGCGAGGGCGTTAATAC
	Reverse	GGGGTAGGTCATGGCATTGA
FBXW7	Forward	GTGATAGAACCCCAGTTTCA
	Reverse	CTTCAGCCAAAATTCTCCAG
ARID1A	Forward	CAGTAAGGGAGGGCAAGAAG
	Reverse	GAGGAGAGAAAGGAGACTGA
CTNNB1	Forward	TCTGAGGACAAGCCACAAGATTACA
	Reverse	TGGGCACCAATATCAAGTCCAA
NOTCH1	Forward	CAGCCTCAACATCCCCTACAAG
	Reverse	GCAGCCCACGAAGAACAGAA
NOTCH2	Forward	GTGGATGGGGTCAACACTTACA
	Reverse	CACTCCAGCCGTTGACACATAC
NOTCH3	Forward	CGTGGCTTCTTTCTACTGTGC
	Reverse	CGTTCACCGGATTTGTGTCAC
NOTCH4	Forward	AACTCCTCCCCAGGAATCTG
	Reverse	CCTCCATCCAGCAGAGGTT
GAPDH	Forward	GAAGGTCGGAGTCAACGGATTT
	Reverse	ATGGGTGGAATCATATTGGAAC

Supplements Table S1 (continued) Primer sequences used in RT-qPCR and MSP experiments.

MSP primers

**Gene**	**Primer**	**Sequence (5‘→ 3‘)**
ALX4	Methylated F	GTTAGGTATGAATGTTGAGATTTGC
	Methylated R	GAATCCCTATACTTTAACGACGAC
	Nonmethylated F	TAGGTATGAATGTTGAGATTTGTGT
	Nonmethylated R	CAAATCCCTATACTTTAACAACAAC
CDX2	Methylated F	CGAAAATAAATCACTACGACG
	Methylated R	AAAGGATATTGGAGAGTATTTTAG
	Nonmethylated F	ATTCAAAATAAAAATCACTACAACA
	Nonmethylated R	AAAGGATATTGGAGAGTATTTTAG
HOPX	Methylated F	GCGAATTAGGAGGTTGAGGTC
	Methylated R	TCGAAACCAAATCTCCGTAACT
	Nonmethylated F	TTGGTGAATTAGGAGGTTGAGGTT
	Nonmethylated R	TCAAAACCAAATCTCCATAACTTCA
ARID1A	Methylated F	GGCGTAGGTTTTAGAGATGC
	Methylated R	AAACGAACTCGCTCCCTT
	Nonmethylated F	GGTGTAGGTTTTAGAGATGT
	Nonmethylated R	AAACAAACTCACTCCCTT

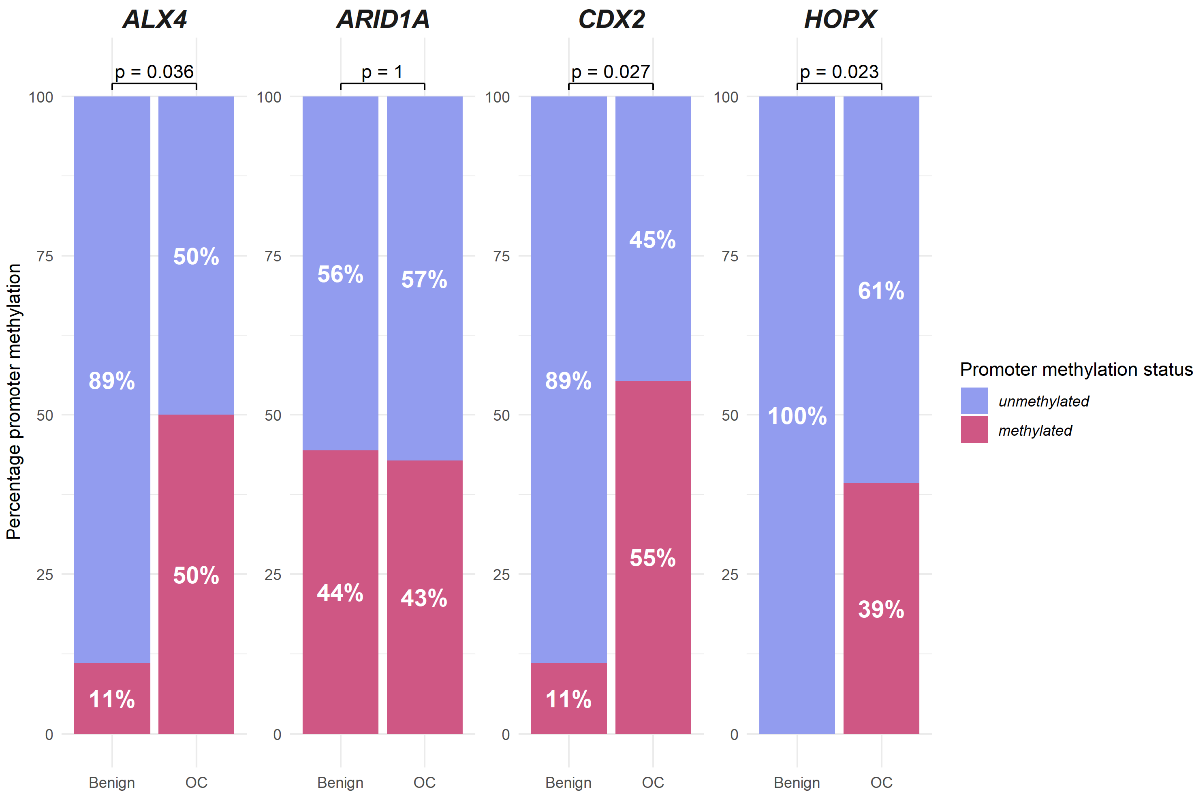

Supplementary Figure 1 *HOX*-related (*ALX4*, *HOPX*, *CDX2*) and *ARID1A* gene promoter methylation in ovarian cancer (OC, n = 56) and benign (n = 9) cases.

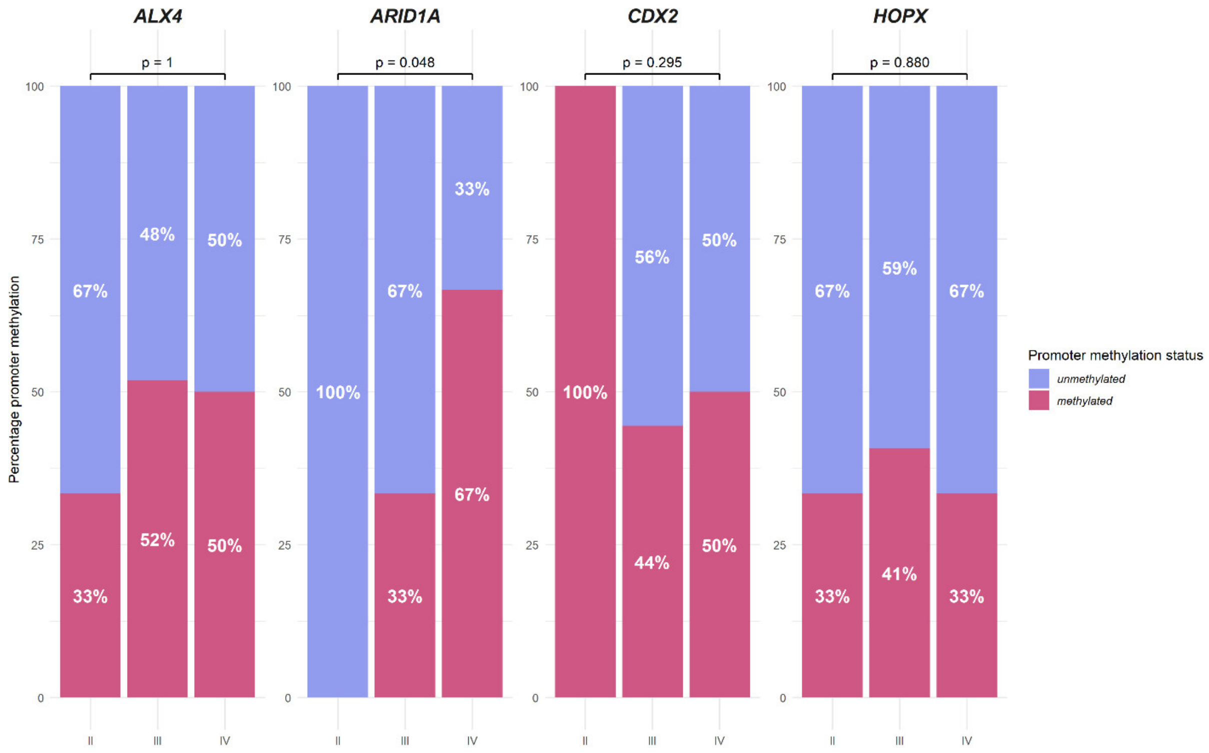

Supplementary Figure 2 *HOX*-related (*ALX4*, *HOPX*, *CDX2*) and *ARID1A* gene promoter methylation in high-grade serous ovarian cancer (HGSOC) group in relation to FIGO stage group (II – Stage 2, n = 3, III – Stage 3, n = 27, IV – Stage 4, n = 12).

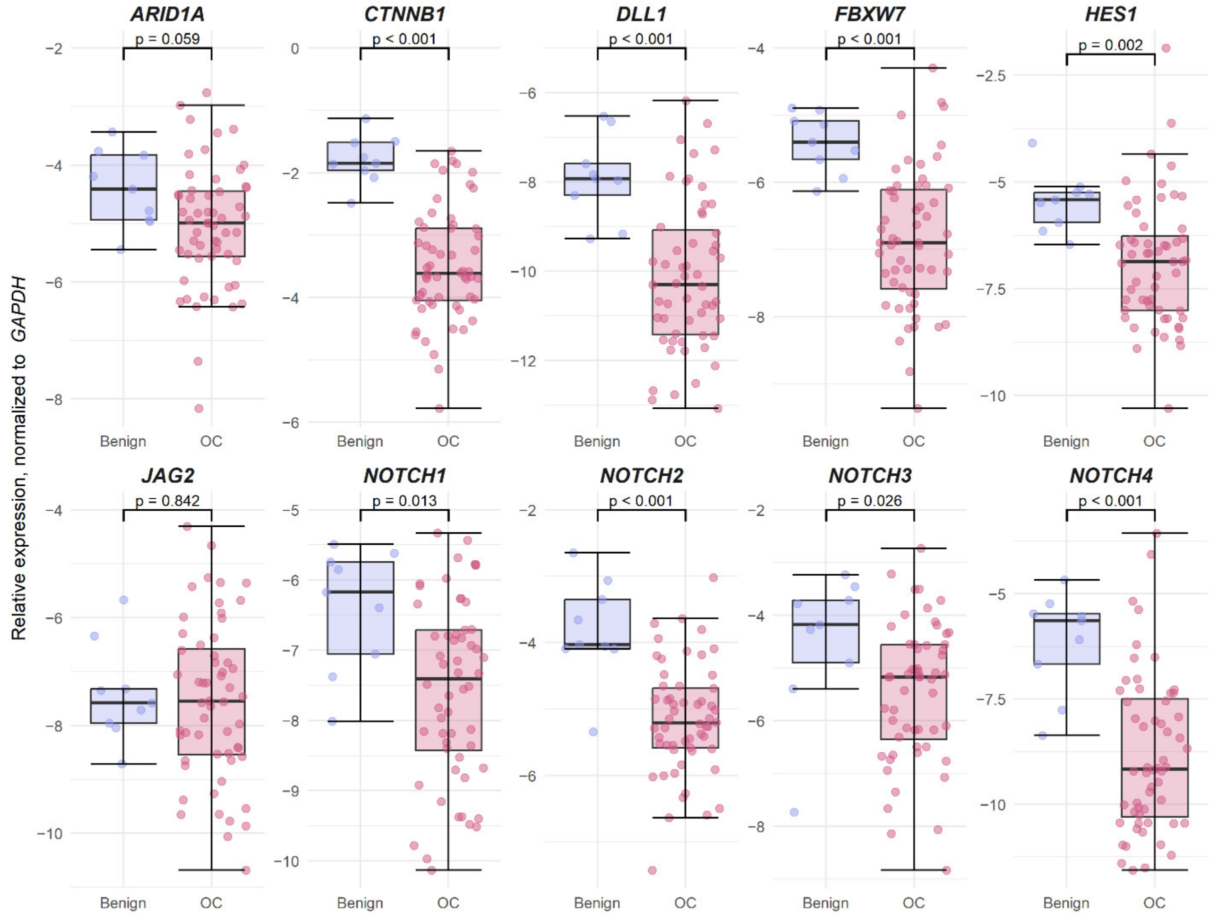

Supplementary Figure 3 Notch pathway related (*DLL1*, *HES1*, *JAG2*, *NOTCH1*, *NOTCH2*, *NOTCH3*, *NOTCH4*), Wnt pathway related (*CTNNB1* and *FBXW7*) and *ARID1A* gene expression in ovarian cancer (OC, n = 56) and benign (n = 9) cases.

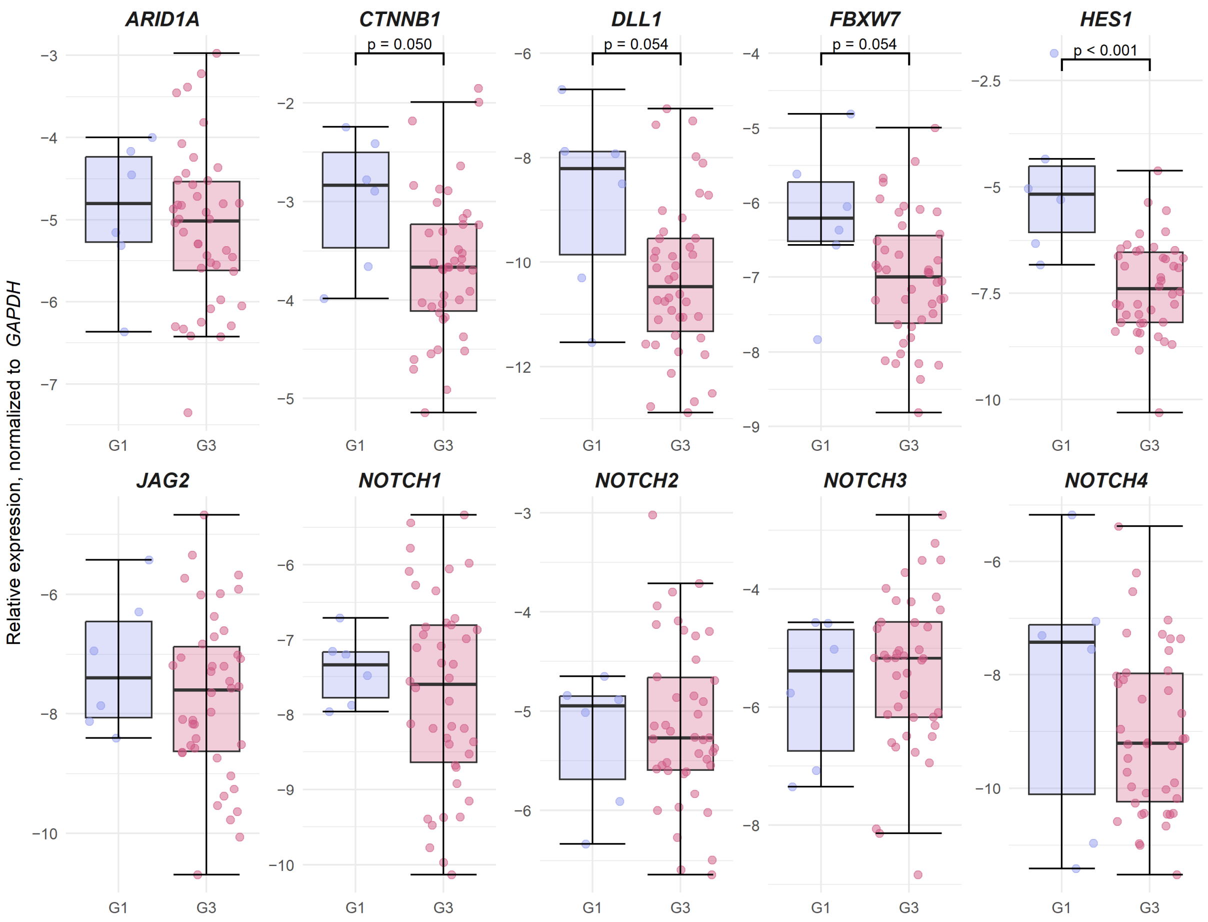

Supplementary Figure 4 Boxplots depicting gene expression in gynecologic tumors in relation to grade group (G1 – grade 1, n = 6; G3 – grade 3, n = 42).

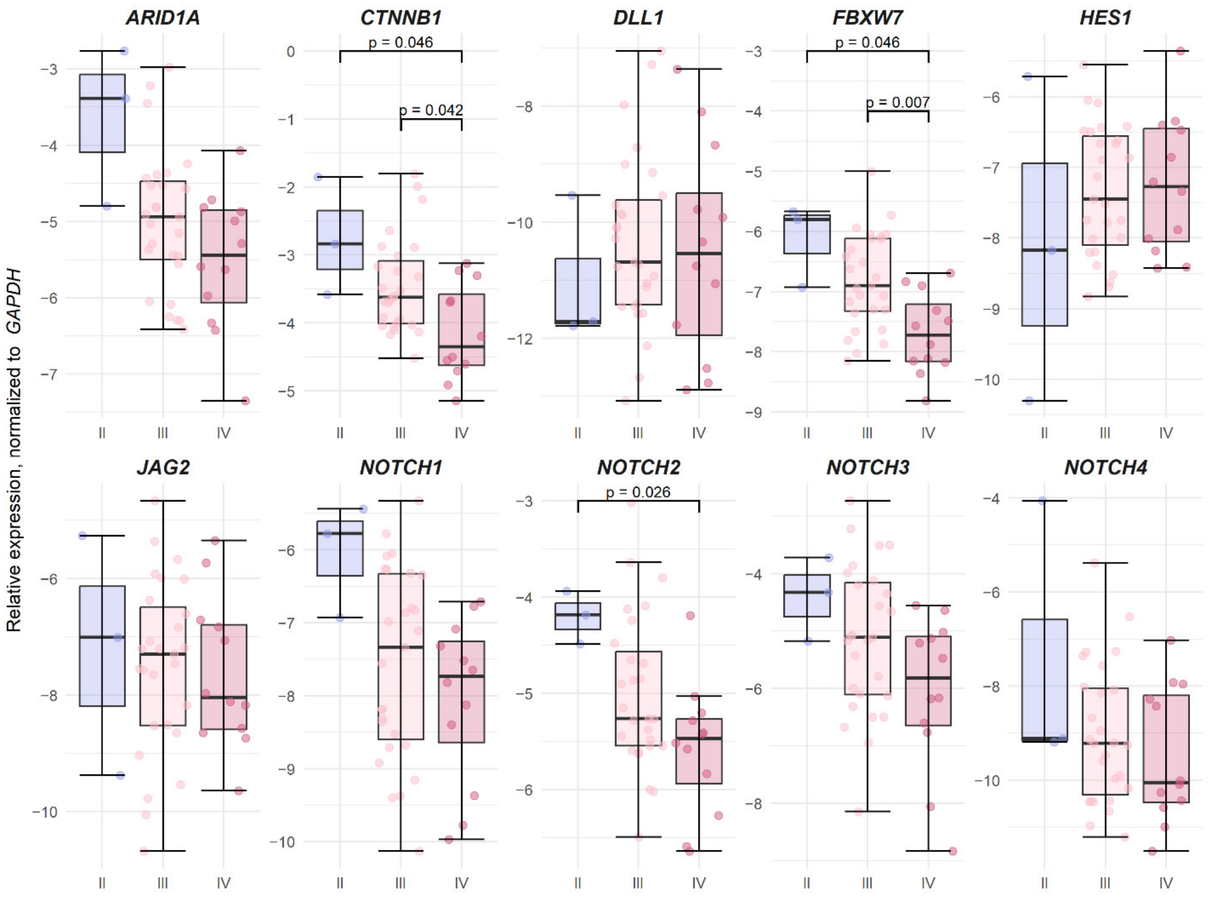

Supplementary Figure 5 Boxplots depicting gene expression in HGSOC group in relation to FIGO stage group (II – Stage 2, n = 3, III – Stage 3, n= 27, IV – Stage 4, n=12).

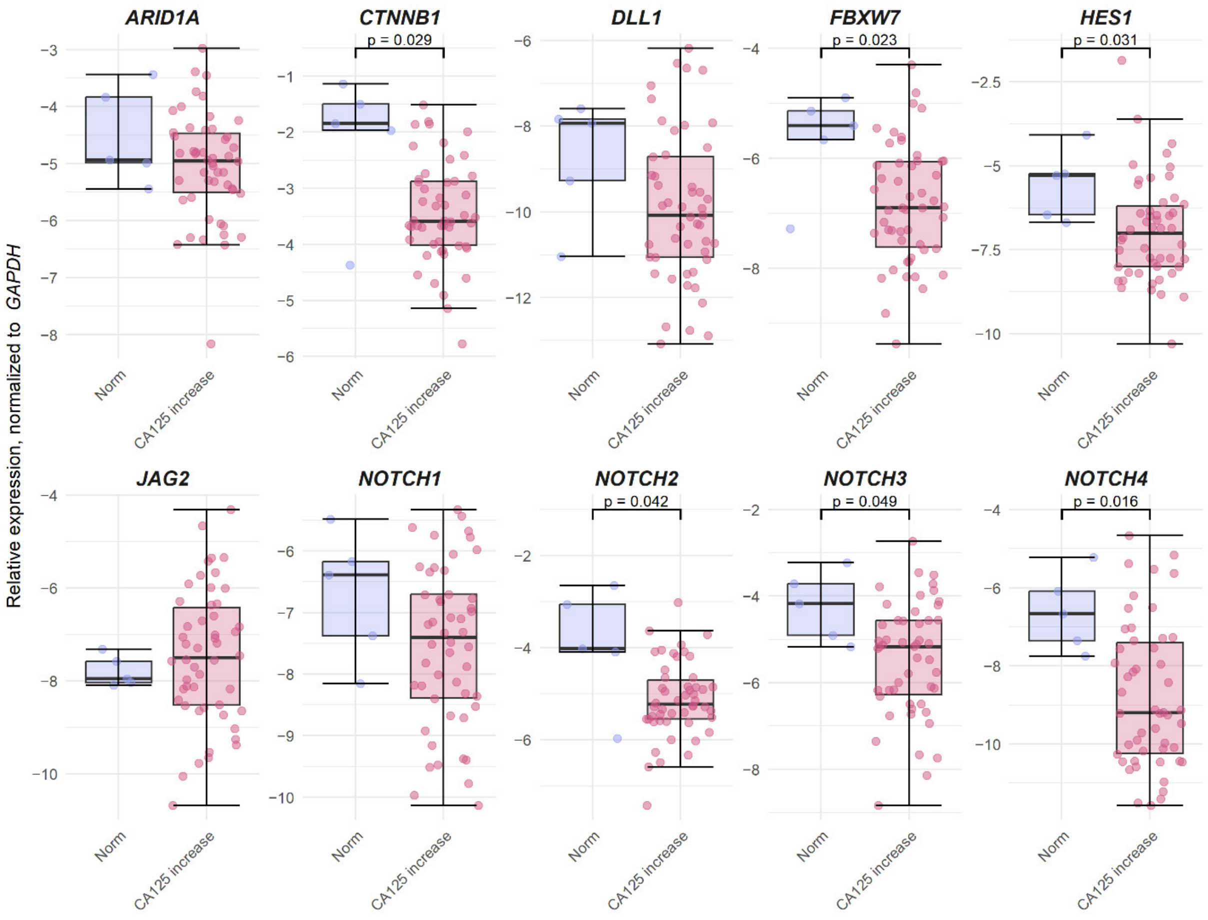

Supplementary Figure 6 Boxplots depicting gene expression in gynecologic tumors in association with CA125 serum concentration status (increased CA125 > 35 U/mL, n = 50 *vs* Norm, n = 5).

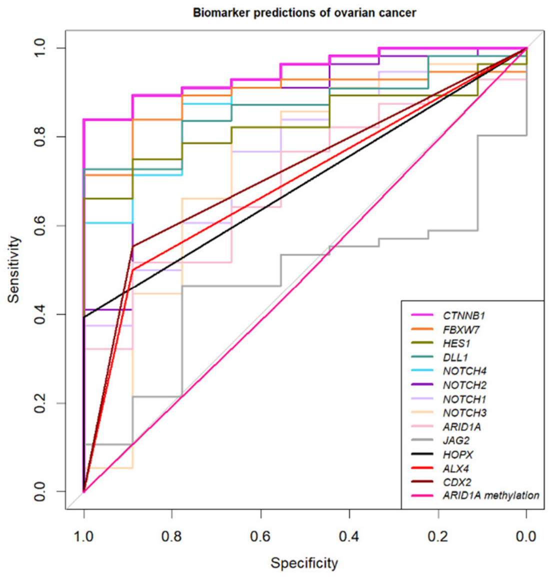

ROC metrics for biomarker predictions of ovarian cancer

Predictor	AUC	accuracy	sensitivity	specificity	precision	npv	tpr	fpr
NOTCH1	0.762	0.754	0.768	0.667	0.935	0.316	0.768	0.333
NOTCH2	0.885	0.892	0.893	0.889	0.980	0.571	0.893	0.111
NOTCH3	0.734	0.800	0.821	0.667	0.939	0.375	0.821	0.333
NOTCH4	0.869	0.862	0.875	0.778	0.961	0.500	0.875	0.222
ARID1A	0.698	0.569	0.518	0.889	0.967	0.229	0.518	0.111
CTNNB1	0.946	0.862	0.839	1.000	1.000	0.500	0.839	0.000
FBXW7	0.893	0.846	0.839	0.889	0.979	0.471	0.839	0.111
JAG2	0.478	0.508	0.464	0.778	0.929	0.189	0.464	0.222
DLL1	0.869	0.766	0.727	1.000	1.000	0.375	0.727	0.000
HES1	0.831	0.708	0.661	1.000	1.000	0.321	0.661	0.000
HOPX	0.696	0.477	0.393	1.000	1.000	0.209	0.393	0.000
ALX4	0.694	0.554	0.500	0.889	0.966	0.222	0.500	0.111
CDX2	0.721	0.600	0.554	0.889	0.969	0.242	0.554	0.111
ARID1A methylation	0.492	0.446	0.429	0.556	0.857	0.135	0.429	0.444

Supplementary Figure 7 Promoter methylation and gene expression biomarkers separation of the ovarian cancer (n = 56) and benign cases (n = 9). AUC – area under the curve, npv – negative predictive value, tpr – true positive rate, fpr – false positive rate.

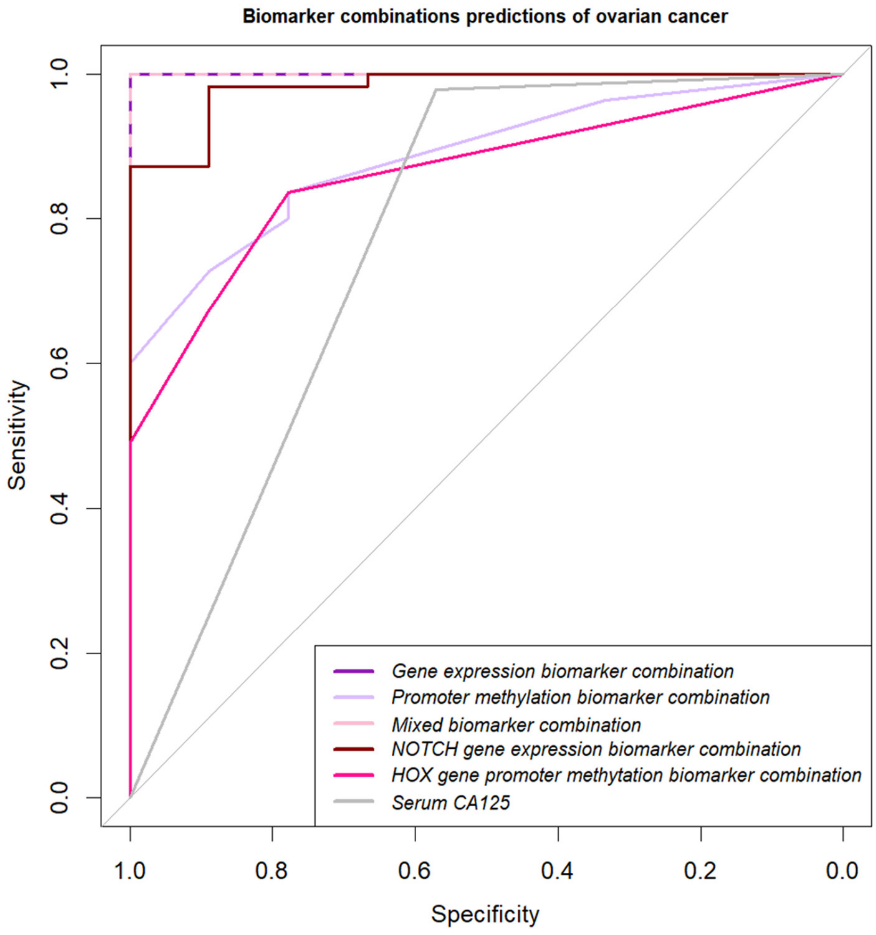

ROC metrics for biomarker combinations predicting ovarian cancer

Predictor	AUC	accuracy	sensitivity	specificity	precision	npv	tpr	fpr
Gene expression biomarker combination	1.000	1.000	1.000	1.000	1.000	1.000	1.000	0.000
Promoter methylation biomarker combination	0.886	0.828	0.836	0.778	0.958	0.438	0.836	0.222
Mixed biomarker combination	1.000	1.000	1.000	1.000	1.000	1.000	1.000	0.000
NOTCH gene expression biomarker combination	0.982	0.969	0.982	0.889	0.982	0.889	0.982	0.111
HOX gene promoter methytation biomarker combination	0.863	0.828	0.836	0.778	0.958	0.438	0.836	0.222
Serum CA125	0.775	0.926	0.979	0.571	0.939	0.800	0.979	0.429

Supplementary Figure 8 ROC plot and its metrics for the gene expression and promoter methylation biomarker combination and serum CA125 biomarker separation of the ovarian cancer (n = 55, one case is missing due to missing *DLL1* expression result) and benign cases (n = 9). Gene expression biomarker combination (all 10 genes), and mixed biomarker combination (all 14 biomarkers) curves overlap. *NOTCH* gene expression biomarker combination includes 7 genes (*NOTCH1-4*, *HES1*, *DLL1*, *JAG2*), *HOX* gene promoter methylation biomarker combination includes 3 genes (*HOPX*, *ALX4*, *CDX2*), serum CA125 threshold concentration considered 35 U/mL.
